# The Suitability of the Childhood Trauma Questionnaire in Criminal Offender Samples

**DOI:** 10.3390/ijerph20065195

**Published:** 2023-03-15

**Authors:** Vera Maria Wente, Petra Retz-Junginger, Anselm Crombach, Wolfgang Retz, Steffen Barra

**Affiliations:** 1 Institute for Forensic Psychology and Psychiatry, Saarland University Hospital, 66421 Homburg, Germanywolfgang.retz@uks.eu (W.R.); 2 Department of Psychology, Saarland University, 66123 Saarbruecken, Germany; 3 Department of Psychiatry and Psychotherapy, University Medical Center, Johannes Gutenberg-University, 55131 Mainz, Germany

**Keywords:** childhood maltreatment, adverse childhood experiences, ACE, bias, delinquency, trauma, reliability

## Abstract

Adverse childhood experiences (ACEs) are common in community samples and are associated with various dysfunctional physical, psychological, and behavioral consequences. In this regard, criminal offenders are at specific risk, considering their elevated ACE rates compared with community samples and the associations of ACEs with criminal behaviors. However, assessing ACEs in offender samples by self-reports has been criticized with regard to their validity and reliability. We examined the suitability of ACE-self-reports using the Childhood Trauma Questionnaire (CTQ) in a sample of 231 male offenders involved in the German criminal justice system by comparing self-reported to externally rated ACEs to externally rated ACEs based on the information from the offenders’ criminal and health-related files and on interviews conducted by forensically trained psychological/psychiatric experts. The accordance between self-ratings and expert ratings was examined considering mean differences, correlations, inter-rater agreement measures, and regression analyses. Offenders themselves reported a higher ACE burden than the one that was rated externally, but there was a strong relationship between CTQ self-assessments and external assessments. However, associations were stronger in offenders seen for risk assessment than in those evaluated for criminal responsibility. Overall, the CTQ seems suitable for use in forensic samples. However, reporting bias in self-reports of ACEs should be expected. Therefore, the combination of self-assessments and external assessments seems appropriate.

## 1. Introduction

In forensic psychology and psychiatry, the assessment and evaluation of offenders are highly important tasks. Judicial decisions based on this information can have far-reaching consequences for sentencing and penitentiary. Within the German penal system, offenders can be assessed, for example, for their criminal responsibility (i.e., can a person be held responsible for his or her actions at the time of the offense?) or risk assessment (e.g., early release from prison). In both cases, the information provided by offenders is of high personal relevance as it can greatly influence their future lives. Hence, clinical experts need to consider the possibility that different assessment settings might influence the answers of criminal offenders.

The role of adverse childhood experiences (ACEs) has gained specific interest when examining the development and maintenance of delinquent behavior. Despite the high number of studies on ACEs, there is no empirical consensus on what experiences to include under this concept [1]. Apart from some certain experiences that are most frequently examined as ACEs (e.g., physical, emotional, and sexual abuse as well as physical and emotional neglect), there is a wide variety of extensions that also include further maladaptive intra- and/or extra-familial dynamics, such as mental illness, illegal drug use, or criminality of family members, interparental violence and separation, or bullying in the peer context [2,3]. Additionally, using the terms “trauma” or “traumatic experiences” poses the risk of confusion with concepts of clinical (psychological) trauma, e.g., in the framework of post-traumatic stress disorder as defined by official diagnostic classification manuals (e.g., DSM-5 [4]). 

International studies have shown, e.g., that individuals affected by severe physical violence prior to the age of 15 years had an increased risk of becoming violent offenders themselves [5]. In line with these results, criminal offenders are particularly likely to report ACEs. Baglivio and colleagues [6] found in a sample of 64,329 juvenile offenders in the U.S. that they were 13 times less likely to report no ACEs at all but 4 times more likely to report 4 or more ACEs than individuals in a community ACE study by Felitti et al. [2]. According to a study of young offenders in Germany, 85% experienced at least one ACE, and more than a quarter of offenders (26.3%) experienced four or more types of ACEs [7]. Moreover, research from Switzerland indicated that 75% of imprisoned juvenile offenders reported more than one ACE during childhood [8]. A study examining the association between ACEs and levels of adult criminal behavior in 338 sentenced adult prison inmates in the U.S. found significantly elevated reports of experienced sexual (31.5%), physical (65.4%), and emotional (56.2%) abuse, as well as emotional (53.8%) and physical (50.3%) neglect [9].

As research indicates a dose—response relationship between ACE exposure and the probability of dysfunctional consequences [2,10], it might be assumed that offender populations are particularly affected by the negative impact associated with ACEs. In addition to an elevated risk of developing mental health disorders [11], high rates of substance abuse and academic/vocational difficulties have been reported, e.g., by Perez and colleagues [12] in a sample of juvenile offenders. Furthermore, they observed that the accumulation of ACEs predicted the expression of impulsivity and aggressiveness. In fact, all of the above-mentioned factors turned out to be predictors of severe, violent, and chronic delinquency [12]. These results confirm prior findings about the impact of ACEs on later delinquent behavior, associating more frequent child abuse with higher rates of delinquency and particularly linking the experience of ACEs to more severe forms of crime (e.g., [13]).

Since the experience of ACEs can have a strong impact on mental health including substance abuse, ACEs are highly relevant when evaluating offenders for criminal responsibility. According to the German Criminal Code (StGB), someone has acted without or with reduced culpability when the person—at the time of the commission of the act—was incapable of (1) recognizing the wrongfulness of the act or (2) acting according to this insight because of a specific mental disorder, a profound disturbance of consciousness, or a severe intellectual disability. As some of these characteristics, especially mental and behavioral disorders (including substance use problems that can foster a disturbance of consciousness), can be influenced, among other things, by the negative effects of ACEs, ACEs need to be taken into account when evaluating offenders for criminal responsibility.

In addition, the consideration of ACEs in forensic psychological/psychiatric risk assessment is particularly relevant because the risk of re-offending appears to increase as a result of the frequent experience of ACEs [14,15]. Several studies have shown that delinquent youth who had experienced ACEs were significantly more likely to recidivate than those who had not experienced ACEs [16,17]. In particular, neglect and physical abuse were significantly associated with the risk of criminal recidivism [15,18]. A meta-analysis by Cottle et al. [19] found a weak but significant association between ACEs and the likelihood of criminal recidivism as well. Baglivio and Epps [20] emphasized an indirect association between an elevated ACE score and an increased risk of criminal recidivism in a sample of nearly 13,000 offenders. Other studies found that the association of ACEs with criminal recidivism was partly mediated by substance use and mental health problems [21]. Some studies even suggested that incorporating ACEs may influence the predictive quality of common criminal risk assessment instruments [22]. Therefore, as part of a comprehensive risk assessment, ACEs should also be recorded in a manner as differentiated as possible [23].

In the context of ACE assessment, the Childhood Trauma Questionnaire (CTQ) ranks among the most frequently used international retrospective instruments; the CTQ covers ACEs on five subscales: physical, emotional, and sexual abuse as well as physical and emotional neglect [24,25,26]. Referring to the abovementioned variety of ACEs, it must be pointed out that - although the name of the CTQ includes the term “trauma” - it does not refer to experiences that necessarily qualify as traumatic in a narrower clinical sense. In order to prevent potential confusion and also to consider the diversity of the ACE concept, we decided to refer to the experiences assessed by the CTQ as ACEs, not trauma or traumatic. First published in 1997, Bernstein and colleagues [25] later developed a shortened version of the CTQ (CTQ-SF). The validity was determined using therapists’ external assessments: For a subsample of psychiatric patients (*n* = 179), therapists were asked to provide detailed information about their patients’ ACEs using a specific interview approach. The therapists had been educated about different types and definitions of ACEs and had received a summary of each patient case based on medical records. However, they had not been informed about their patients’ CTQ self-ratings. Inter-rater reliability was excellent. The five CTQ subscales (self-reports) significantly predicted the therapists’ ratings [25].

The CTQ has been used on several occasions in forensic settings. When applied among 338 adult offenders, the CTQ was reported to be a highly reliable and valid retrospective instrument for assessing ACEs [9]. Despite its original development for adults, its successful use with delinquent youth has also been documented [11,18,27,28]. The five-factor structure was largely confirmed by factor analysis in a sample of 272 delinquent adolescents [18].

However, the quality of CTQ results has also been criticized because of its nature as a retrospective self-assessment tool. Brown et al. [29] published a longitudinal study in which they compared ACE self-assessments of 644 adolescents in New York State with official data from the Central Registry of Child Abuse and Neglect. They concluded that in most cases data did not match. There were both cases of overreporting (respondents reported more ACEs than were recorded in the official files), which the authors explained by the fact that the rate of unreported child abuse is very high, and cases of underreporting (respondents reported fewer ACEs than were recorded in the official files), which the authors explained, for example, by the fact that the youth did not understand the abuse definitions or did not want to remember them. Moreover, differences might emerge based on the assessment procedure—i.e., self-report questionnaires vs. clinician-administered interviews. Interviews tended to yield more ACEs than questionnaires [30,31]. Nevertheless, people appreciated the use of questionnaires due to greater perceived anonymity, especially when reporting rather intimate experiences, such as physical and sexual abuse [32,33].

Further criticism of retrospective ACE self-assessment includes the potential for distorted reports due to recall bias [34] or underreporting due to shame or fear of stigmatization [11]. Susser and Widom [35] summarized a variety of arguments why data on ACEs based solely on individuals’ self-assessments are prone to error: (a) false memory can develop during recall (see also [36]); (b) recall performance depends on the respondents’ memory performance and motivation to recall; (c) memories that go back a long time are likely to be influenced by information given in the meantime (e.g., by parents, other caregivers, or therapists); (d) ACEs are associated with amnesia and other types of forgetting (see also [37,38]); (e) statements about ACEs are influenced by the current emotional state (see also [39]). There could also be a difference in recall of ACEs between older and younger individuals, following the above points, as older individuals’ ACEs were further in the past than younger individuals’ ACEs and, therefore, might be less easily recalled [40,41]. Mental illness, particularly of an affective nature, has also been associated with biased memory performance [42,43]. Furthermore, the influence of psychotherapeutic interventions as a possible source of bias in self-assessments of ACEs has also been discussed. Some authors have hypothesized that successful therapeutic treatment would make ACEs less likely to be reflected in individuals’ statements, as ACEs would no longer be associated with previous distressing emotions [44]. Others have stressed that ACEs would be strengthened by replay in the therapeutic setting and thus be better retrieved [33].

Some additional aspects need to be taken into consideration when assessing ACEs in criminal offender samples. Some offenders might overreport ACEs in self-assessments when trying to justify their actions and/or trying to provoke sympathy by stressing their role as a victim. Moreover, offenders might consider their own child abuse and neglect histories as more relevant if they have been convicted of a violent and/or sexual crime themselves instead of some other (nonviolent/sexual) criminal act [45]. However, underreporting of ACEs in the forensic setting might also happen. When offenders are told that their information will be used for clinical purposes or criminal assessment, the results might be biased by a perceived lack of confidentiality [40]. Some offenders might try to present themselves as less affected to avoid placement in a psychiatric institution. Such a placement often renders the actual length of treatment/containment uncertain and might entail an additional feeling of stigmatization [46]. Moreover, it might seem advantageous for offenders to downplay their ACE exposure during risk assessment hoping to be released early from prison.

For these reasons, the use of the CTQ in offender populations is in urgent need of review to analyze the extent to which a questionnaire whose data are based on respondents’ retrospective self-assessments can provide reliable results when offenders are aware that their responses might have an impact on their further criminal proceedings. To the best of our knowledge, a possible discrepancy between self-assessment and external assessment of ACEs in a sample of delinquent individuals has not yet been investigated. Furthermore, the impact of the context of assessment (evaluation of criminal responsibility or risk of criminal recidivism) on ACE self-reports has never been examined.

To address this gap in the research, the present study compared the CTQ self-reports of criminal offenders with corresponding external ACE assessments based on psychological/psychiatric evaluation reports and considered potential differences depending on the context of assessment (evaluation of criminal responsibility or risk of criminal recidivism). We assumed that the CTQ self-assessment would be at least moderately and positively associated with the CTQ external assessment. Considering previous research [11,34,35,37,39,40,42,43,45], it was hypothesized that the strength of agreement would differ depending on the context of assessment (evaluation of criminal responsibility or risk of criminal recidivism).

## 2. Materials and Methods

### 2.1. Study Design and Procedure

Data were obtained within an ongoing retrospective analysis of psychological and psychiatric evaluation reports at the Institute for Forensic Psychology and Psychiatry, Homburg, Germany. Since May 2020, we have analyzed data from a consecutive sample of criminal offenders who were seen at the Institute for psychological/psychiatric evaluations between August 2007 and February 2020. Evaluations were based on information from the offenders’ criminal and health-related files and on personal interviews with the offenders conducted by forensically trained psychiatrists and psychologists. Interviews included questions about biographical information (including family dynamics and education), physical, mental, and sexual development, history of health problems (including substance abuse), as well as former and current delinquency. Moreover, offenders were asked to complete several self-rating instruments (e.g., assessing personality and aggression, but also including CTQ-SF).

The final evaluation reports were analyzed by trained psychologists and psychiatrists who had not been involved in the basic evaluation process using a structured coding scheme [47], which was adapted for use in adult offenders based on a previous scheme already proven in studies on criminal file analyses in juvenile offender samples [48,49,50]. On 44 pages, the coding scheme included forensically relevant variables assigned to the following areas (see also [51]): (1) administrative data (e.g., name, date), (2) demographic information (e.g., place of residence, country of origin), (3) current/index delinquency (e.g., type of offense, victim characteristics), (4) offense analysis (e.g., degree of violence, alcohol/drugs involved), (5) previous delinquency (e.g., criminal records), (6) biographic/family information (e.g., childhood residential area, family diseases), (7) general and sexual development (e.g., school career, romantic relationships, also previous and current psychiatric diagnoses), (8) adverse childhood experiences (e.g., different types of intra- and extrafamilial abuse and neglect), (9) the content of forensic evaluation (e.g., the profession of the evaluator, type of evaluation: risk assessment vs. criminal responsibility), and (10) risk assessment (e.g., considered risk assessment instruments). To determine inter-rater reliability, 30 randomly selected evaluations (stratified for the context of assessment: criminal responsibility vs. risk of criminal recidivism) were independently double-rated. Study procedures were approved by the ethics committee of the Medical Chamber of Saarland, Germany (protocol code: No. 179/21).

### 2.2. Participants

A total of 235 evaluations were coded at the time of the present study, including 131 (55.7%) on criminal responsibility, 100 (42.6%) on risk assessment, and 4 (1.7%) on other questions (e.g., the inability of arrest). Since the present study focused on the comparison of criminal responsibility vs. risk evaluations, the four other cases were excluded. The remaining 231 evaluations included offenders between 16 and 73 years of age at the time of their evaluations (M = 36.33 years, SD = 11.92 years). Offenders evaluated for criminal responsibility were significantly younger (M = 33.27 years, SD = 11.32 years) than offenders evaluated for risk assessment (M = 39.91 years, SD= 11.68 years), *t*(209.71) = −4.34, *p* < 0.001. A total of 108 offenders (46%) had committed at least one violent crime, 60 (25.5%) offenders had committed a sexual crime and 67 (28.5%) offenders had committed other (nonviolent/nonsexual) crimes (e.g., theft, fraud, or drug offenses). In addition, lifetime behavioral/psychiatric disorders (i.e., psychotic, affective, personality disorders, ADHD, or substance use disorders) were more frequent among offenders evaluated for criminal responsibility (M = 1.76, SD = 1.27) than among offenders evaluated for risk assessment (M = 0.73, SD = 1.00), χ² (6) = 46.52, *p* < 0.001, *V* = 0.45 (see Supplementary Materials, Table S1).

### 2.3. Measurements

The extent of ACEs up to the age of 18 years was assessed by the 25-item CTQ-SF [25], which included 5 items per subscale (emotional abuse, physical abuse, sexual abuse, emotional neglect, and physical neglect). As mentioned above, offenders conducted the self-rating version of the CTQ-SF as part of the evaluation process. For CTQ-SF external ratings, the eighth section of the above-mentioned coding scheme contained the equivalent questions of the CTQ-SF; however, adapted from self-rating to external rating by changing the subject of each question from first-person singular to third-person singular. The external raters had no knowledge of the offenders’ self-reports in the CTQ-SF. Items were rated on a 5-point Likert scale ranging from 1 (“never”) to 5 (“very often”). A total sum score and the subscale scores were built, with higher scores representing more frequent and/or more severe maltreatment.

The inter-rater reliability for the sum score and all subscale scores of the CTQ-SF for external assessment was excellent (ICC = 0.89–0.94). Apart from the physical neglect subscale, the CTQ-SF demonstrated good reliability and validity in previous studies [26]. In the present study, Cronbach’s α for the entire sample (self-assessment as well as external assessment) indicated excellent internal consistency for the sum score and all subscale scores except for the physical abuse subscale, which showed acceptable consistency (see Table 1 and Supplementary Materials, Table S2).

For descriptive purposes, we also considered information on lifetime psychiatric disorders extracted from the evaluation reports. Reports included diagnoses determined by the psychological or psychiatric expert who conducted the evaluation as well as former diagnoses that were listed in past medical records.

### 2.4. Statistical Analyses

Data were analyzed using the open-source software R-Studio [52]. The general level of significance was set at *p* = 0.05. Cronbach’s α measured internal consistency, with α ≥ 0.60 representing questionable, α ≥ 0.70 acceptable, α ≥ 0.80 good, and α ≥ 0.90 excellent consistency [53]. Inter-rater reliability for external CTQ-SF ratings was examined by the intra-class correlation coefficient (ICC). Mean differences between the CTQ-SF self-assessments and external assessments were calculated using two-sample t-tests for dependent samples. As an effect size measure, Cohen’s *d* was considered using interpretation cut-offs of *d* = 0.20 (small effect), *d* = 0.50 (medium effect), and *d* = 0.80 (large effect). To detect possible group differences regarding mental health between the offenders evaluated for criminal responsibility vs. those evaluated for criminal risk, a Chi-squared test was carried out. Cramer’s V and Phi coefficient (ϕ) were used as effect sizes and evaluated using the following cutoffs: ϕ/V = 0.10 (small effect), ϕ/V = 0.30 (medium effect), ϕ/V = 0.50 (large effect) [54].

To quantify the strength of the association between the CTQ–SF internal and external assessments, the Pearson product-moment correlation was calculated with the corresponding 95% confidence intervals (CI) with coefficient cutoffs interpreted according to Cohen (1988): small correlation |*r*| = 0.10, medium correlation |*r*| = 0.30, and large correlation |*r*| = 0.50. The absolute agreement between the CTQ-SF self-assessment and external assessment was additionally checked by calculating the ICC [55]. We used the function *ICC()* of the package *DescTools* (version 0.99.44) with its variant *ICC2* (two-way random effects, single rater, absolute agreement; see [55]). ICC < 0.50 indicated poor, ICC ≥ 0.50 moderate, ICC ≥ 0.75 good, and ICC ≥ 0.90 excellent reliability [55]. We further assessed whether the type of evaluation led to a bias of the general values by conducting a two-sample Welch’s test for independent samples, which is robust to lack variance homogeneity. Cohen’s *d* was used to interpret effect sizes (see above).

To examine whether the type of evaluation had a significant effect on the relationship between the CTQ-SF self-assessment and external assessment, we first examined whether the correlation coefficients, which described the association between self-assessments and external assessments, differed significantly between the responsibility vs. risk group using the function *paired.r*() from the r package *psych* (Version number 2.2.5, W. Revelle, Northwestern University, Evanston, Illinois, USA; https://CRAN.R-project.org/package=psych). Second, we conducted a stepwise multiple linear regression analysis using the CTQ-SF external ratings as an outcome [25] and including interaction terms between CTQ-SF self-assessment and context of evaluation (criminal responsibility vs. risk).

Age at the time of psychological/psychiatric evaluation and intelligence (based on clinical evaluation and/or psychometric testing by the respective assessor) were included as control variables to counteract bias due to possible differences in memory quality [40,41], cohort effects [26], differences in susceptibility to suggestibility [56], or performance of working, long-term [57], and episodic memory [58]. Independent variables were mean-centered to facilitate the interpretation of any main effects that might occur [59]. To avoid inflation of the type 1 error due to multiple testing, the Bonferroni—Holm correction [60] was applied.

## 3. Results

### 3.1. Differences between Self and External Assessment

As shown in Table 1, the CTQ-SF self-assessment scores were significantly higher than the external assessment scores for the sum score as well as for emotional abuse, physical abuse, sexual abuse, and physical neglect. A self-assessment lower than the external rating was only observed for the emotional neglect subscale. The small effects remained significant after applying the Bonferroni—Holm correction.

### 3.2. Associations between Self and External Assessment

Table 2 shows that CTQ-SF self-assessment and external assessment scores were strongly correlated regarding the sum score and all subscale scores. Absolute agreement between self-reports and external reports in the overall sample was good for the sum score and the emotional abuse, physical abuse, sexual abuse, and emotional neglect subscales. Physical neglect showed a moderate absolute agreement (see Table 3 and Supplementary Materials, Table S3).

### 3.3. Differences and Associations between Self-Assessment and External Assessment within Subgroups

Within the group of offenders evaluated for criminal responsibility, we found a similar pattern as in the overall sample: CTQ-SF self-assessment scores were significantly higher than external report scores regarding the sum score and the emotional abuse, physical abuse, sexual abuse, and physical neglect subscale scores. Only the emotional neglect subscale showed self-assessment scores lower than external assessment scores, but the difference was not significant (see Table 4). After applying the Bonferroni—Holm correction, the mean difference regarding physical abuse was no longer significant.

We found a strong correlation between self-reports and external reports for the sum score as well as for all subscale scores within the group of offenders evaluated for criminal responsibility (see Table 4 and Supplementary Materials, Table S4). The sum score and the subscale scores regarding emotional and physical abuse as well as emotional and physical neglect showed a moderate absolute agreement between self-assessment and external assessment, whereas the sexual abuse subscale showed good absolute agreement (see Supplementary Materials, Table S6).

Within the group of offenders evaluated for criminal risk, self-assessment scores were significantly higher than external assessment scores only for the sum score as well as physical abuse and neglect. For emotional and sexual abuse, the self-assessment scores were also higher than external assessment scores, but the differences were not significant. The self-assessment emotional neglect score was lower than the external assessment score, but the difference was not significant either. Effects were mostly weak. Moderate effects were only found for physical neglect in both groups. The Bonferroni—Holm correction did not affect the results (see Table 5).

As already seen in the group of offenders evaluated for criminal responsibility, we observed a strong correlation between self-assessment and external assessment for the sum score and all subscale scores as well in the group of offenders evaluated for risk assessment (see Table 5 and Supplementary Materials, Table S5). The sum score as well as sexual abuse and emotional neglect subscale scores showed a good absolute agreement between self-assessment and external assessment, whereas emotional abuse, physical abuse, and physical neglect scores showed a moderate absolute agreement (see Supplementary Materials, Table S6).

### 3.4. Differences and Associations between Self-Assessment and External Assessment Depending on the Context of Evaluation

Offenders who had completed the CTQ-SF as part of a risk assessment tended to report higher scores than those who had completed the CTQ-SF as part of an evaluation of criminal responsibility. This tendency was found for the sum score and for all subscale scores except emotional neglect. However, only the difference in physical neglect was significant (see Table 6).

After applying the Bonferroni—Holm correction, no difference remained significant. In addition, offenders who were assessed as part of a responsibility evaluation reported higher scores than those who were seen for risk assessment. However, only the difference in physical abuse ratings was significant. After applying the Bonferroni—Holm correction, no difference remained significant (see Table 7).

Table 8 presents the comparisons of correlation coefficients, which describe the associations of self-assessment and external assessment within the groups of offenders evaluated for criminal responsibility vs. risk. The correlations in the risk group tended to be stronger than in the criminal responsibility group. However, only the correlations of the sum scores and the sexual abuse and emotional neglect subscales differed significantly. The correlation of self-assessment and external assessment of the sum score and of the emotional neglect subscale in the risk group was significantly higher than in the criminal responsibility group, whereas the correlation of sexual abuse subscales was lower in the risk group than in the criminal responsibility group.

**Figure 1:**
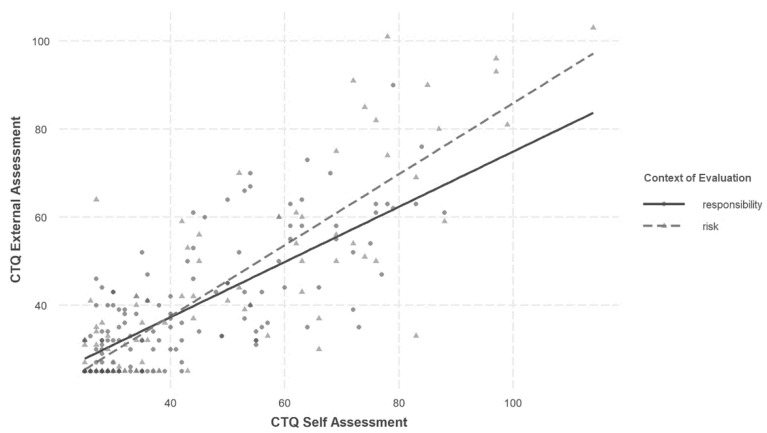
Relationship between CTQ self-assessment and external assessment of the sum score depending on the context of evaluation.

Running a stepwise multiple linear regression with interaction revealed that there was a main effect of the independent variable CTQ-SF self-assessment for the sum score and all subscale scores (see Supplementary Materials, Table S7). Adding the interaction term (self-assessment × context of evaluation) as a predictor led to a significant increase in the explained variance in the sum score, *F*(1, 229) = 6.07, *p* = 0.015, and the emotional abuse subscale scores, *F*(1, 229) = 5.65, *p* = 0.018. This increase in variance remained significant even after adding the control variables of age and intelligence. For all other subscale scores, the interaction term showed no significant effect. Thus, the positive predictive effect of self-assessment on external assessment is stronger for the sum and emotional abuse score for the risk group than for the criminal responsibility group (see Figure 1 and Supplementary Materials, Table S7).

## 4. Discussion

### 4.1. General Findings

The aim of the present study was to compare the CTQ-SF self-assessment and external assessment in a criminal offender sample in order to examine its applicability in forensic psychology and psychiatry. In addition, we examined whether the context of forensic evaluation (criminal responsibility vs. risk assessment) significantly influenced the relationship between self-reports and external reports.

A significant mean difference between the self-assessment and external assessment of the total sample was evident for the sum score as well as for almost all subscale scores. Offenders rated their ACEs more severely than externally rated on all subscales except emotional neglect, although the effects were small. Most likely, the unstructured assessment regarding childhood maltreatment during the interviews and the use of official sources of information, e.g., health records and medical reports, in the preparation of the evaluations may have missed a considerable number of unreported ACEs, especially less severe forms of ACEs. This effect may have resulted in a lower external assessment compared with a self-assessment, which would align with research findings indicating that informant-based assessments yield lower prevalence rates [61].

Furthermore, offenders might have been tempted to justify their own criminal acts or to portray themselves as less responsible for their behaviors by reporting rather frequent exposure to ACEs. If they themselves had been convicted of (child) maltreatment, this may have led them to recall their own ACEs as more formative than when they were externally judged [45]. Decreased external assessment compared with self-assessment may have also resulted from the fact that the confidentiality of the interview was limited due to the appraisal situation resulting in offenders reporting fewer details [40]. Possibly, differences between self-assessment and external assessment may have occurred when some experiences were asked for the first time through the self-report questionnaire and these ACEs were not mentioned in personal interviews, e.g., due to shame or fear of stigmatization [11].

Despite small mean score differences, CTQ-SF self-assessments and external assessments correlated strongly for the sum score as well as for all subscales, which is in line with the results of Bernstein and colleagues [25]. The lowest correlation was found for the physical neglect subscale, which is consistent with previous findings that attributed poor reliability to this scale [26]. Moderate to good agreement scores (ICC) on the sum and subscale scores further confirmed the results, with agreement also being lowest for physical neglect. Studies examining the psychometric quality of the German version of the CTQ-SF found reduced item discriminatory power and reduced internal consistency for the physical neglect subscale compared with the other subscales and attributed these findings to the ambiguous wording of some items of this scale. They argued that the content of those items could also be assigned to the emotional neglect or emotional abuse subscale [62]. Klinitzke and colleagues [26] found a significant age effect for some items, and they suspected a cohort effect as the cause for the insufficient reliability of the physical neglect subscale. According to Klinitzke et al. [26], increased scores on the physical neglect subscale in older subjects could be due to deprivation after World War II (see also [63]) and, thus, would not be dependent on the family context but on historical circumstances.

As in the overall sample, offenders within the two groups (evaluated for criminal responsibility vs. risk assessment) rated themselves as more burdened than when they were rated externally, although the effects were predominantly small. Within groups, the responsibility group showed more significant mean differences between self-assessment and external assessment scores than the risk group. In the responsibility group, the mean scores of the sum score, as well as the subscale scores on emotional abuse, physical abuse, sexual abuse, and physical neglect differed significantly whereas, in the risk group, significant mean differences occurred only for the sum score and physical abuse and physical neglect subscales.

The results of the present study are rather contrary to the assumption that offenders evaluated for risk assessment may be motivated to paint a picture of themselves that is as stable and unencumbered as possible (see also [39]) in order to create certain advantages for the further course of prosecution, e.g., to increase their chances of being released from prison. In addition, within the risk group, self-assessment scores were higher than external assessment scores overall, so there was no evidence of underreporting among offenders in this sample. The correlations between self-assessment and external assessment within each group were predominantly strong.

A possible age effect in the sense of reduced recall performance with respect to ACEs with increasing age [40,41] could not be demonstrated based on the present data. Since the offenders in the risk group were significantly older than those in the responsibility group and the relevant memories were further in the past; a reduced memory performance should have led to a higher discrepancy between self-assessment and external assessment in the risk group, which, however, was not the case in the present study.

Regarding the potential effects of the evaluation context (criminal responsibility vs. risk assessment) on self-reports and external reports of ACEs, offenders in the responsibility group tended to report fewer ACEs than those in the risk group. It could be that at this point—compared with the risk group—they tended to deal less with offense reappraisal (e.g., in the context of a therapeutic treatment) and their past, and thus, no therapy effect in terms of better accessibility and reflectivity (see also [33]) could occur. Another factor that may have contributed to lower self-reports of offenders evaluated for criminal responsibility is that a feeling of stigmatization often accompanies the incapacity and the placement in psychiatric institutions (see also [46]), and thus, offenders may have tried to present themselves as unencumbered as possible for fear of this stigmatization [11]. In addition, the subjects may have been more willing to present themselves as psychologically stable in order to avoid a psychiatric placement, unpredictable in terms of duration. However, these assumptions are only partially reflected in the data, as the self-assessment of the responsibility group was not significantly lower compared with the risk group.

CTQ self-assessment and external assessments tended to be correlated more strongly in the risk group than in the responsibility group. This difference was significant for the sum score and the emotional neglect subscale. However, the sexual abuse subscale showed a significantly higher correlation in the responsibility group than in the risk group. The ICC also indicated higher agreement in the risk group, although the 95% CIs overlapped significantly. The multiple linear regression results suggest that the association of self-assessment and external assessment is significantly stronger in the risk group than in the responsibility group for the sum score and the emotional abuse subscale.

The significantly stronger correlation of self-assessment and external assessment on most scales in the risk group suggests that there tend to be somewhat fewer bias tendencies or discrepancies between self-assessment and external assessment in this context of evaluation. The results are consistent with previous findings that suggest that stronger psychological adversity, such as is present in the responsibility group e.g., due to affective disorders, may be reflected in a weaker relationship between self-assessment and external assessment [42,43].

Two findings deviated from the general pattern. First, the emotional neglect subscale was the only scale in the overall sample to show no significant mean difference between self-assessment and external assessment. This scale, in particular, might have been expected to show particularly low external assessment, since this form of ACE seldom appears in official records (see also [18]). Quite contrary to this assumption, emotional neglect was the most frequently reported type of ACEs in both self-assessment and external assessment compared with the other subscales. It is possible that the items of the emotional neglect subscale, which were sometimes difficult to distinguish from other items, e.g., of the emotional abuse and physical neglect subscales (see also [26,62]), were answered rather liberally by raters because the majority of offenders showed an adverse developmental history, which might have increased the estimation of emotional neglect from the raters’ perspective. Offenders’ adverse developmental experiences in combination with the somewhat indistinct formulation of some emotional neglect items could have led to high self-assessment scores too.

Sexual abuse was the only subscale for which the correlation between self and external assessment was higher in the responsibility group than in the risk group. It might be that the offenders in this group, e.g., due to a lack of therapeutic reprocessing, had lower accessibility to these rather shameful ACEs and, therefore, disclosed particularly little information in the questionnaire as well as in the personal interview, which could have led to a tendency toward lower self-assessment and external assessment. Many sexual abuse experiences are not reported by victims because of shame, fear of consequences, or lack of accurate memories [64]. Accordingly, information from official records may also underestimate the actual prevalence of sexual abuse, which may also have contributed to the low external assessment score, resulting in the strong association of self-assessment and external assessment in the responsibility group. Therapeutic reappraisal of childhood experiences might have made offenders in the risk group more willing to disclose sexually abusive experiences [33], which could have led to increased self-assessment. However, external assessment could still be affected by underreporting in case files and in face-to-face interviews (e.g., due to shame [11] or lack of confidentiality [40]), which would explain the lower association of self-assessment and external assessment in the risk group compared with the responsibility group. The dynamics underlying the discrepancies or whether those were statistical artifacts could not be conclusively clarified in the current study.

### 4.2. Strengths and Limitations

#### 4.2.1. The Sample Characteristics

The present study is based on a consecutive sample of offenders that can be considered comparatively large for the forensic psychological/psychiatric context and heterogeneous with respect to age and committed offenses. However, although the sample used here is heterogeneous for the forensic domain, offenders represent a rather homogeneous group compared with the general population, which may have resulted in high internal consistencies on the CTQ reports. Furthermore, the sample consists exclusively of men who were seen at one specific institute in Germany. The generalizability of the findings is thus limited. In particular, gender-specific effects demonstrated in other studies, which indicate that the associations between ACEs and delinquency may be stronger for men than women [65], could not be examined. Moreover, studies on these relations among people of nonbinary gender identity are still missing. Furthermore, as discussed in the present paper, offenders’ responses may have been influenced by specific biases due to the juridical context. Regarding the associations of ACE self-reports and external reports, it remains unclear whether similar results would occur in a non-forensic general population sample.

#### 4.2.2. Study Design and Procedures

Two groups of offenders were cross-sectionally compared according to the context of evaluation (criminal responsibility vs. risk assessment) in the present study. A longitudinal study design, which could have compared the data of the same offenders in both initial responsibility evaluation and subsequent risk assessment, could have provided further information to explain possible reporting bias.

The use of well-established procedures for evaluation by forensic psychological and psychiatric experts and the coding of the assessments by trained staff ensured high data quality. Since this was a correlative study and the construct of ACE is very complex, a confounding influence of third-party variables could not be ruled out completely even with the use of proven procedures and conscientious evaluation. However, inter-rater reliability was rated excellent for all subscales and the sum score of the CTQ-SF for external assessment. With regard to ACE self-assessment, it should be noted that the three items of the CTQ-SF, which are intended to detect minimization and denial, were not included in the original test battery used at the time of evaluation and, thus, were not available for analysis in the present study, although they could have provided further information for the interpretation of the results [66,67].

#### 4.2.3. Specifics of the CTQ

The CTQ(-SF) itself has some strengths and limitations that should always be considered when using it. The CTQ as well as the CTQ-SF were found to be useful in the general population and clinical samples [62,68,69], as well as in forensic samples [6,9,14,18]—the latter supported by the present study. The frequent application of the CTQ-SF is, among other things, due to its high cost-effectiveness. The short processing time of about five minutes allows its integration into test batteries without much additional effort. Second, the narrow definition of the five types of ACEs makes it possible to compare and analyze data across multiple studies [70]. Meta-analyses were often guided by the five types of ACEs interrogated by the CTQ-SF, as these were covered in most studies (see also [71]). However, the five-factor structure of the CTQ, as postulated by Bernstein et al. [24], has been repeatedly criticized [26,72,73,74,75].

The CTQ has been translated into many different languages and its psychometric quality has been repeatedly reviewed (see [72,76,77]). A study that systematically matched 52 child abuse measurement instruments from a total of 2095 studies with criteria from the COSMIN (COnsensus-based Standards for the selection of health status Measurement INstruments) checklist [78], described the CTQ as the only instrument that was “thoroughly investigated” and demonstrated “a strong level of evidence with adequate internal consistency, reliability, content validity, structural validity and convergent [hypothesis testing] validity” [71]. The long version of the CTQ performed best of all the methods examined. It met 55% of the COSMIN scale criteria with moderate to strong evidence, followed by the CTQ-SF and the Maltreatment and Abuse Chronology of Exposure (MACE) scale [3], both of which met 44% of the criteria with moderate to strong evidence [71].

The high economy of the instrument is inevitably accompanied by some limitations. For example, the CTQ does not provide a chronological classification. Items refer to childhood in general, but neither the exact onset of ACEs nor their duration is recorded. However, this temporal classification seems relevant because the negative consequences of ACEs may vary depending on the onset and duration of ACEs [79]. Depending on whether or not ACEs fall within a critical developmental period, they may have different effects on the development of psychopathologies [80,81] and also the course of criminal careers [48]. Saini et al. [71] emphasize the use of the MACE scale over the CTQ for temporal classification of ACEs, which was developed for this very purpose and captures not only exposure time but also cumulative severity and the number of types of experienced ACEs.

Moreover, although most frequently examined, experiences of emotional, physical, and sexual abuse as well as emotional and physical neglect only represent a small selection of ACEs [82]. Other childhood adversity types, such as peer assault or witnessing domestic violence [3,49,83,84], are also common and have been associated with increased risk for psychopathology and criminogenic development [3,22,82]. However, these types are not covered by the narrow definition of ACEs in the CTQ or CTQ-SF. Again, the MACE scale may serve as a promising alternative as it includes 10 types of ACEs.

## 5. Conclusions

The high level of agreement between the CTQ-SF self-assessment and external assessment in the present study suggests that the CTQ-SF self-report is suitable for use in samples of criminal offenders. However, some bias should be expected. Our findings show that offenders achieved higher ACE scores in self-reports than in external ratings. As deviations from self-assessment and external assessment occurred less frequently within the offenders evaluated for risk assessment than within the offenders evaluated for criminal responsibility, the use of the CTQ-SF in criminal responsibility evaluations tends to be associated with more uncertainty than in risk assessments.

Longitudinal research is needed to further examine which bias tendencies might be causal for the discrepancy between self-assessment and external assessment. Including the denial scale of the CTQ could provide additional insights [66]. When investigating the reliability of retrospective self-reports on ACEs, comparisons with official records appear beneficial, although less severe forms of ACEs are likely to be underrepresented in case files [33]. Thus, a combination of self-reports, current case files, and prior developmental documentation (e.g., youth welfare reports) is recommended [29]. To narrow down underlying causes of bias in ACE reports in forensic evaluations, offenders should complete the CTQ prior to the interview. Thus, psychological or psychiatric experts can directly mention possible discrepancies between self-reports and external information in personal dialogue while addressing potential feelings of shame, stigmatization, or perceived lack of confidentiality [11,40].

## Figures and Tables

**Table 1:** Descriptive Statistics and Mean Differences between CTQ-SF Self- and External Assessment.

CTQ-SF	M (SD)		*t*(230)	95% CI	*p*	*d*	Cronbach’s α	
	Self	External					Self	External
Sum	44.41(19.00)	40.62 (17.38)	4.94	[2.28, 5.30]	<0.001	0.325	0.95	0.96
EA	9.30 (5.18)	8.16 (4.91)	4.30	[0.62, 1.66]	<0.001	0.283	0.96	0.93
PA	8.16 (4.94)	7.18 (4.07)	4.11	[0.50, 1.44]	<0.001	0.270	0.94	0.96
SA	5.81 (2.86)	5.55 (2.25)	2.31	[0.04, 0.47]	0.022	0.152	0.96	0.98
EN	11.50 (6.02)	11.71 (5.84)	−0.69	[−0.80, 0.39]	0.493	0.045	0.94	0.74
PN	9.65 (4.02)	8.01 (3.62)	7.34	[1.19, 2.07]	<0.001	0.482	0.74	0.77

Note. CI = confidence interval, sum = sum score, EA = emotional abuse, PA = physical abuse, SA = sexual abuse, EN = emotional neglect, PN = physical neglect, self = self-assessment, external = external assessment. Tests were conducted two-sided.

**Table 2:** Pearson Correlation Coefficients of CTQ Self-Assessment and External Assessment in the Total Sample.

CTQ-SF	*r*	95% CI
Sum	0.80 **	[0.75, 1.00]
EA	0.68 **	[0.61, 1.00]
PA	0.70 **	[0.64, 1.00]
SA	0.81 **	[0.76, 1.00]
EN	0.70 **	[0.64, 1.00]
PN	0.61 **	[0.54, 1.00]

Note. *N* = 231. CI = confidence interval, sum = sum score, EA = emotional abuse, PA = physical abuse, SA = sexual abuse, EN = emotional neglect, PN = physical neglect. Tests were conducted one-sided. ** *p* ≤ 0.01.

**Table 3:** Absolute Agreement between CTQ Self-Assessment and External Assessment in the Total Sample.

CTQ-SF		95% CI					
	ICC	LL	UL	*F*	df1	df2	*p*
Sum	0.78	0.70	0.84	8.80	230	230	<0.001
EA	0.67	0.57	0.74	5.30	230	230	<0.001
PA	0.67	0.58	0.74	5.30	230	230	<0.001
SA	0.78	0.73	0.83	8.40	230	230	<0.001
EN	0.70	0.63	0.76	5.60	230	230	<0.001
PN	0.56	0.38	0.69	4.10	230	230	<0.001

Note. *N* = 231, Number of judges = 2. ICC = Intra-class correlation coefficient; CI = confidence interval; LL = lower limit, UL = upper limit; Sum = sum score, EA = emotional abuse, PA = physical abuse, SA = sexual abuse, EN = emotional neglect, PN = physical neglect.

**Table 4:** Mean Differences between CTQ Self-Assessment and External Assessment within the Criminal Responsibility Group.

CTQ-SF	*r*	M (SD)		*t*(130)	*p*	*d*
		Self	External			
Sum	0.74	43.26 (16.64)	39.34 (14.11)	3.95	<0.001	0.345
EA	0.64	9.02 (4.81)	7.60 (3.97)	4.27	<0.0001	0.373
PA	0.64	7.55 (4.26)	6.63 (3.23)	3.16	0.002	0.276
SA	0.88	5.56 (2.00)	5.34 (1.59)	2.51	0.013	0.219
EN	0.62	11.54 (5.74)	11.79 (5.47)	−0.57	0.568	0.050
PN	0.57	9.60 (4.02)	7.98 (3.33)	5.34	<0.001	0.467

Note. *n* = 131. Sum = sum score, EA = emotional abuse, PA = physical abuse, SA = sexual abuse, EN = emotional neglect, PN = physical neglect, self = self-assessment, external = external assessment. Tests were conducted two-sided.

**Table 5:** Mean Differences between CTQ Self-Assessment and External Assessment within the Risk Assessment Group.

CTQ-SF	*r*	M (SD)		*t*(99)	*p*	*d*
		Self	External			
Sum	0.84	45.92 (21.71)	42.29 (20.88)	2.996	0.004	0.300
EA	0.72	9.68 (5.64)	8.9 (5.85)	1.809	0.074	0.180
PA	0.73	8.95 (5.64)	7.9 (4.88)	2.651	<0.001	0.265
SA	0.78	6.14 (3.68)	5.83 (2.89)	1.343	0.182	0.134
EN	0.78	11.44 (6.39)	11.6 (6.31)	−0.379	0.705	0.038
PN	0.67	9.71 (4.05)	8.06 (3.99)	5.027	<0.001	0.503

Note. *n* = 100. Sum = sum score, EA = emotional abuse, PA = physical abuse, SA = sexual abuse, EN = emotional neglect, PN = physical neglect, self = self-assessment, external = external assessment. Tests were conducted two-sided.

**Table 6:** Comparison of the Mean Self-Assessment Scores between the Criminal Responsibility (*n* = 131) and Risk Assessment (*n* = 100) Groups.

CTQ-SF	M (SD)		*t*	*df*	*p*	95% CI
	Criminal Responsibility	Risk Assessment				
Sum	43.26 (16.64)	45.92 (21.71)	−1.018	180.08	0.310	[−7.82, 2.49]
EA	9.02 (4.81)	9.68 (5.64)	−0.946	193.96	0.345	[−2.05, 0.72]
PA	7.55 (4.26)	8.95 (5.64)	−2.072	178.11	0.040	[−2.73, −0.67]
SA	5.56 (2.00)	6.14 (3.68)	−1.431	143.18	0.155	[−1.39, 0.22]
EN	11.54 (5.74)	11.44 (6.40)	0.126	200.41	0.901	[−1.50, 1.70]
PN	9.60 (4.02)	9.71 (4.05)	−0.214	212.36	0.831	[−1.17, 0.94]

Note. Sum = sum score, EA = emotional abuse, PA = physical abuse, SA = sexual abuse, EN = emotional neglect, PN = physical neglect, CI = confidence interval. Tests were conducted two-sided.

**Table 7:** Comparison of the Mean External Assessment Scores between the Criminal Responsibility (*n* = 131) and Risk Assessment (*n* = 100) Groups.

CTQ-SF	M (SD)		*t*	*df*	*p*	95% CI
	Criminal Responsibility	Risk Assessment				
Sum	39.34 (14.11)	42.29 (20.88)	−1.215	164.81	0.226	[−7.73, 1.84]
EA	7.60 (3.97)	8.90 (5.85)	−1.906	165.36	0.058	[−2.64, 0.05]
PA	6.63 (3.23)	7.90 (4.88)	−2.247	162.39	0.026	[−2.38, −0.15]
SA	5.34 (1.59)	5.83 (2.89)	−1.517	143.96	0.132	[−1.20, 0.15]
EN	11.79 (5.47)	11.6 (6.31)	0.235	196.00	0.814	[−1.38, 1.75]
PN	7.98 (3.33)	8.06 (4.00)	−0.168	191.28	0.867	[−1.06, 0.89]

Note. Sum = sum score, EA = emotional abuse, PA = physical abuse, SA = sexual abuse, EN = emotional neglect, PN = physical neglect, CI = confidence interval. Tests were conducted two-sided.

**Table 8:** Comparison of Pearson Correlation Coefficients of CTQ Self-Assessment and External Assessment between the Criminal Responsibility (*n* = 131) and Risk Assessment (*n* = 100) Groups.

CTQ-SF	Criminal Responsibility		Risk Assessment		*z*	*p*
	*r*	95% CI	*r*	95% CI		
Sum	0.74	[0.65, 0.81]	0.84	[0.77, 0.89]	1.99	0.050
EA	0.64	[0.53, 0.73]	0.72	[0.61, 0.80]	1.05	0.290
PA	0.64	[0.52, 0.73]	0.73	[0.62, 0.81]	1.23	0.220
SA	0.88	[0.83, 0.91]	0.78	[0.69, 0.85]	2.37	0.020
EN	0.62	[0.50, 0.72]	0.78	[0.69, 0.85]	2.37	0.020
PN	0.57	[0.44, 0.67]	0.67	[0.54, 0.76]	1.18	0.240

Note. Sum = sum score, EA = emotional abuse, PA = physical abuse, SA = sexual abuse, EN = emotional neglect, PN = physical neglect. Tests were conducted two-sided.

## Data Availability

The data presented in this study are available on request from the last author (S.B.).

## Supplementary Materials

The following supporting information can be downloaded at: https://www.mdpi.com/article/10.3390/ijerph20065195/s1, Table S1: Numbers of Lifetime Mental Disorders Depending on the Context of Evaluation. Table S2: Cronbach´s α of the Childhood Trauma Questionnaire Depending on the Context of Evaluation. Table S3: Correlations of Childhood Trauma Questionnaire Self and External Assessment in the Total Sample (*N* = 231). Table S4: Correlations of Childhood Trauma Questionnaire Self and External Assessment in Offenders Evaluated for Criminal Responsibility (*n* = 131). Table S5: Correlations of Childhood Trauma Questionnaire Self and External Assessment in Offenders Evaluated for Risk Assessment (*n* = 100). Table S6: Intra-class Correlation Coefficient between Self and External Assessment within the Groups Criminal Responsibility and Risk Assessment. Table S7: Stepwise Linear Regression with Interaction to Predict the External Assessment of the Childhood Trauma Questionnaire.

## References

[1] Kalmakis K.A., Chandler G.E. (2015). Health Consequences of Adverse Childhood Experiences: A Systematic Review. J. Am. Assoc. Nurse Pract..

[2] Felitti V.J., Anda R.F., Nordenberg D., Williamson D.F., Spitz A.M., Edwards V., Koss M.P., Marks J.S. (1998). Relationship of Childhood Abuse and Household Dysfunction to Many of the Leading Causes of Death in Adults: The Adverse Childhood Experiences (ACE) Study. Am. J. Prev. Med..

[3] Teicher M.H., Parigger A. (2015). The ‘Maltreatment and Abuse Chronology of Exposure’ (MACE) Scale for the Retrospective Assessment of Abuse and Neglect During Development. PLoS ONE.

[4] American Psychiatric Association (2013). Diagnostic and Statistical Manual of Mental Disorders (DSM-5).

[5] Webb R.T., Antonsen S., Carr M.J., Appleby L., Pedersen C.B., Mok P.L. (2017). Self-Harm and Violent Criminality among Young People Who Experienced Trauma-Related Hospital Admission during Childhood: A Danish National Cohort Study. Lancet Public Health.

[6] Baglivio M.T., Epps N., Swartz K., Sayedul Huq M., Sheer A., Hardt N.S. (2013). The Prevalence of Adverse Childhood Experiences (ACE) in the Lives of Juvenile Offenders. J. Juv. Justice.

[7] Barra S., Turner D., Müller M., Hertz P.G., Retz-Junginger P., Tüscher O., Huss M., Retz W. (2022). ADHD Symptom Profiles, Intermittent Explosive Disorder, Adverse Childhood Experiences, and Internalizing/Externalizing Problems in Young Offenders. Eur. Arch. Psychiatry Clin. Neurosci..

[8] Bielas H., Barra S., Skrivanek C., Aebi M., Steinhausen H.-C., Bessler C., Plattner B. (2016). The Associations of Cumulative Adverse Childhood Experiences and Irritability with Mental Disorders in Detained Male Adolescent Offenders. Child Adolesc. Psychiatry Ment. Health.

[9] Cuadra L.E., Jaffe A.E., Thomas R., DiLillo D. (2014). Child Maltreatment and Adult Criminal Behavior: Does Criminal Thinking Explain the Association?. Child Abuse Negl..

[10] Brown D.W., Anda R.F., Tiemeier H., Felitti V.J., Edwards V.J., Croft J.B., Giles W.H. (2009). Adverse Childhood Experiences and the Risk of Premature Mortality. Am. J. Prev. Med..

[11] Turner D., Wolf A.J., Barra S., Müller M., Gregório Hertz P., Huss M., Tüscher O., Retz W. (2021). The Association between Adverse Childhood Experiences and Mental Health Problems in Young Offenders. Eur. Child Adolesc. Psychiatry.

[12] Perez N.M., Jennings W.G., Baglivio M.T. (2018). A Path to Serious, Violent, Chronic Delinquency: The Harmful Aftermath of Adverse Childhood Experiences. Crime Delinq..

[13] Lansford J.E., Miller-Johnson S., Berlin L.J., Dodge K.A., Bates J.E., Pettit G.S. (2007). Early Physical Abuse and Later Violent Delinquency: A Prospective Longitudinal Study. Child Maltreat..

[14] Baglivio M.T., Wolff K.T., Piquero A.R., Epps N. (2015). The Relationship between Adverse Childhood Experiences (ACE) and Juvenile Offending Trajectories in a Juvenile Offender Sample. J. Crim. Justice.

[15] Van der Put C.E., de Ruiter C. (2016). Child Maltreatment Victimization by Type in Relation to Criminal Recidivism in Juvenile Offenders. BMC Psychiatry.

[16] Cho M., Lee C.H. (2022). Childhood Maltreatment and Repeat Offending in Juvenile Delinquents: A Propensity Score Matched-Control Study. Youth Soc..

[17] Li D., Chu C.M., Goh J.T.L., Ng I.Y.H., Zeng G. (2015). Impact of Childhood Maltreatment on Recidivism in Youth Offenders: A Matched-Control Study. Crim. Justice Behav..

[18] Kingree J.B., Phan D., Thompson M. (2003). Child Maltreatment and Recidivism among Adolescent Detainees. Crim. Justice Behav..

[19] Cottle C.C., Lee R.J., Heilbrun K. (2001). The Prediction of Criminal Recidivism in Juveniles: A Meta-Analysis. Crim. Justice Behav..

[20] Baglivio M.T., Epps N. (2016). The Interrelatedness of Adverse Childhood Experiences Among High-Risk Juvenile Offenders. Youth Violence Juv. Justice.

[21] Craig J.M., Zettler H.R., Wolff K.T., Baglivio M.T. (2019). Considering the Mediating Effects of Drug and Alcohol Use, Mental Health, and Their Co-Occurrence on the Adverse Childhood Experiences–Recidivism Relationship. Youth Violence Juv. Justice.

[22] Barra S., Bessler C., Landolt M.A., Aebi M. (2018). Testing the Validity of Criminal Risk Assessment Tools in Sexually Abusive Youth. Psychol. Assess..

[23] Guarnaccia C., De Vita E., Sortino L., Giannone F. (2022). Links between Adverse Childhood Experiences, Psychopathological Symptoms and Recidivism Risk in Juvenile Delinquents. Eur. J. Criminol..

[24] Bernstein D.P., Ahluvalia T., Pogge D., Handelsman L. (1997). Validity of the Childhood Trauma Questionnaire in an Adolescent Psychiatric Population. J. Am. Acad. Child Adolesc. Psychiatry.

[25] Bernstein D.P., Stein J.A., Newcomb M.D., Walker E., Pogge D., Ahluvalia T., Stokes J., Handelsman L., Medrano M., Desmond D. (2003). Development and Validation of a Brief Screening Version of the Childhood Trauma Questionnaire. Child Abuse Negl..

[26] Klinitzke G., Romppel M., Häuser W., Brähler E., Glaesmer H. (2012). Die deutsche Version des Childhood Trauma Questionnaire (CTQ)–psychometrische Eigenschaften in einer bevölkerungsrepräsentativen Stichprobe. PPmP Psychother. Psychosom. Med. Psychol..

[27] Aebi M., Linhart S., Thun-Hohenstein L., Bessler C., Steinhausen H.-C., Plattner B. (2015). Detained Male Adolescent Offender’s Emotional, Physical and Sexual Maltreatment Profiles and Their Associations to Psychiatric Disorders and Criminal Behaviors. J. Abnorm. Child Psychol..

[28] Carrion V.G., Steiner H. (2000). Trauma and Dissociation in Delinquent Adolescents. J. Am. Acad. Child Adolesc. Psychiatry.

[29] Brown J., Cohen P., Johnson J.G., Salzinger S. (1998). A Longitudinal Analysis of Risk Factors for Child Maltreatment: Findings of a 17-Year Prospective Study of Officially Recorded and Self-Reported Child Abuse and Neglect. Child Abuse Negl..

[30] Diaz A., Peake K., Nucci-Sack A., Shankar V. (2017). Comparison of Modes of Administration of Screens to Identify a History of Childhood Physical Abuse in an Adolescent and Young Adult Population. Ann. Glob. Health.

[31] Martin J., Anderson J., Romans S., Mullen P., O’Shea M. (1993). Asking about Child Sexual Abuse: Methodological Implications of a Two Stage Survey. Child Abuse Negl..

[32] DiLillo D., DeGue S., Kras A., Di Loreto-Colgan A.R., Nash C. (2006). Participant Responses to Retrospective Surveys of Child Maltreatment: Does Mode of Assessment Matter?. Violence Vict..

[33] Hardt J., Rutter M. (2004). Validity of Adult Retrospective Reports of Adverse Childhood Experiences: Review of the Evidence. J. Child Psychol. Psychiatry.

[34] Nanni V., Uher R., Danese A. (2012). Childhood Maltreatment Predicts Unfavorable Course of Illness and Treatment Outcome in Depression: A Meta-Analysis. Am. J. Psychiatry.

[35] Susser E., Widom C.S. (2012). Still Searching for Lost Truths About the Bitter Sorrows of Childhood. Schizophr. Bull..

[36] Sacktor T.C. (2011). How Does PKMζ Maintain Long-Term Memory?. Nat. Rev. Neurosci..

[37] Feldman-Summers S., Pope K.S. (1994). The Experience of “Forgetting” Childhood Abuse: A National Survey of Psychologists. J. Consult. Clin. Psychol..

[38] Williams L.M. (1994). Recall of Childhood Trauma: A Prospective Study of Women’s Memories of Child Sexual Abuse. J. Consult. Clin. Psychol..

[39] Prescott A., Bank L., Reid J.B., Knutson J.F., Burraston B.O., Eddy J.M. (2000). The Veridicality of Punitive Childhood Experiences Reported by Adolescents and Young Adults. Child Abuse Negl..

[40] Boonmann C., Grisso T., Guy L.S., Colins O.F., Mulder E.A., Vahl P., Jansen L.M.C., Doreleijers T.A.H., Vermeiren R.R.J.M. (2016). Childhood Traumatic Experiences and Mental Health Problems in Sexually Offending and Non-sexually Offending Juveniles. Child Adolesc. Psychiatry Ment. Health.

[41] Squire L.R. (1989). On the Course of Forgetting in Very Long-Term Memory. J. Exp. Psychol. Learn. Mem. Cogn..

[42] Dalgleish T., Werner-Seidler A. (2014). Disruptions in Autobiographical Memory Processing in Depression and the Emergence of Memory Therapeutics. Trends Cogn. Sci..

[43] Joormann J., Teachman B.A., Gotlib I.H. (2009). Sadder and Less Accurate? False Memory for Negative Material in Depression. J. Abnorm. Psychol..

[44] Baldwin J.R., Reuben A., Newbury J.B., Danese A. (2019). Agreement Between Prospective and Retrospective Measures of Childhood Maltreatment: A Systematic Review and Meta-Analysis. JAMA Psychiatry.

[45] Aylwin A., Studer L., Reddon J., Clelland S. (2003). Abuse Prevalence and Victim Gender among Adult and Adolescent Child Molesters. Int. J. Law Psychiatry.

[46] Volkow N.D., Gordon J.A., Koob G.F. (2021). Choosing Appropriate Language to Reduce the Stigma around Mental Illness and Substance Use Disorders. Neuropsychopharmacology.

[47] Barra S., Thönnes E. (2020). Strukturiertes Kodierungssystem Für Forensische Gutachten (SKFG).

[48] Barra S., Bessler C., Landolt M.A., Aebi M. (2017). Type and Timing of Maltreatment Influence Criminal Persistence in Sexually Abusive Adolescents. Law Hum. Behav..

[49] Barra S., Bessler C., Landolt M.A., Aebi M. (2018). Patterns of Adverse Childhood Experiences in Juveniles Who Sexually Offended. Sex. Abuse.

[50] Krause C., Roth A., Landolt M.A., Bessler C., Aebi M. (2021). Validity of Risk Assessment Instruments Among Juveniles Who Sexually Offended: Victim Age Matters. Sex. Abuse.

[51] Woehrle L., Retz-Junginger P., Retz W., Barra S. (2022). The Maltreatment–Aggression Link among Prosecuted Males: What about Psychopathy?. Int. J. Environ. Res. Public. Health.

[52] RStudio Team (2022). RStudio: Integrated Development Environment for R.

[53] Blanz M. (2021). Forschungsmethoden und Statistik für die Soziale Arbeit: Grundlagen und Anwendungen.

[54] Cohen J. (1988). Statistical Power Analysis for the Behavioral Sciences.

[55] Koo T.K., Li M.Y. (2016). A Guideline of Selecting and Reporting Intraclass Correlation Coefficients for Reliability Research. J. Chiropr. Med..

[56] Gudjonsson G.H. (1983). Suggestibility, Intelligence, Memory Recall and Personality: An Experimental Study. Br. J. Psychiatry.

[57] Unsworth N. (2010). On the Division of Working Memory and Long-Term Memory and Their Relation to Intelligence: A Latent Variable Approach. Acta Psychol..

[58] Herlitz A., Yonker J.E. (2002). Sex Differences in Episodic Memory: The Influence of Intelligence. J. Clin. Exp. Neuropsychol..

[59] Dalal D.K., Zickar M.J. (2012). Some Common Myths About Centering Predictor Variables in Moderated Multiple Regression and Polynomial Regression. Organ. Res. Methods.

[60] Holm S. (1979). A Simple Sequentially Rejective Multiple Test Procedure. Scand. J. Stat..

[61] Stoltenborgh M., Bakermans-Kranenburg M.J., Alink L.R., van IJzendoorn M.H. (2015). The Prevalence of Child Maltreatment across the Globe: Review of a Series of Meta-analyses. Child Abuse Rev..

[62] Wingenfeld K., Spitzer C., Mensebach C., Grabe H.J., Hill A., Gast U., Schlosser N., Höpp H., Beblo T., Driessen M. (2010). Die deutsche Version des Childhood Trauma Questionnaire (CTQ): Erste Befunde zu den psychometrischen Kennwerten. PPmP Psychother. Psychosom. Med. Psychol..

[63] Häuser W., Schmutzer G., Brähler E., Glaesmer H. (2011). Maltreatment in Childhood and Adolescence. Dtsch. Ärztebl..

[64] Garrett A., Hassan N. (2020). Understanding the Silence of Sexual Harassment Victims through the #WhyIDidntReport Movement. Proceedings of the 2019 IEEE/ACM International Conference on Advances in Social Networks Analysis and Mining.

[65] Leban L., Gibson C.L. (2020). The Role of Gender in the Relationship between Adverse Childhood Experiences and Delinquency and Substance Use in Adolescence. J. Crim. Justice.

[66] Church C., Andreassen O.A., Lorentzen S., Melle I., Aas M. (2017). Childhood Trauma and Minimization/Denial in People with and without a Severe Mental Disorder. Front. Psychol..

[67] MacDonald K., Thomas M.L., Sciolla A.F., Schneider B., Pappas K., Bleijenberg G., Bohus M., Bekh B., Carpenter L., Carr A. (2016). Minimization of Childhood Maltreatment Is Common and Consequential: Results from a Large, Multinational Sample Using the Childhood Trauma Questionnaire. PLoS ONE.

[68] Hagborg J.M., Kalin T., Gerdner A. (2022). The Childhood Trauma Questionnaire—Short Form (CTQ-SF) Used with Adolescents–Methodological Report from Clinical and Community Samples. J. Child Adolesc. Trauma.

[69] Scher C.D., Stein M.B., Asmundson G.J.G., McCreary D.R., Forde D.R. (2001). The Childhood Trauma Questionnaire in a Community Sample: Psychometric Properties and Normative Data. J. Trauma Stress.

[70] Humphreys K.L., LeMoult J., Wear J.G., Piersiak H.A., Lee A., Gotlib I.H. (2020). Child Maltreatment and Depression: A Meta-Analysis of Studies Using the Childhood Trauma Questionnaire. Child Abuse Negl..

[71] Saini S.M., Hoffmann C.R., Pantelis C., Everall I.P., Bousman C.A. (2019). Systematic Review and Critical Appraisal of Child Abuse Measurement Instruments. Psychiatry Res..

[72] Gerdner A., Allgulander C. (2009). Psychometric Properties of the Swedish Version of the Childhood Trauma Questionnaire—Short Form (CTQ-SF). Nord. J. Psychiatry.

[73] Grassi-Oliveira R., Cogo-Moreira H., Salum G.A., Brietzke E., Viola T.W., Manfro G.G., Kristensen C.H., Arteche A.X. (2014). Childhood Trauma Questionnaire (CTQ) in Brazilian Samples of Different Age Groups: Findings from Confirmatory Factor Analysis. PLoS ONE.

[74] Villano C.L., Cleland C., Rosenblum A., Fong C., Nuttbrock L., Marthol M., Wallace J. (2004). Psychometric Utility of the Childhood Trauma Questionnaire with Female Street-Based Sex Workers. J. Trauma Dissociation.

[75] Wright K.D., Asmundson G.J.G., McCreary D.R., Scher C., Hami S., Stein M.B. (2001). Factorial Validity of the Childhood Trauma Questionnaire in Men and Women. Depress. Anxiety.

[76] He J., Zhong X., Gao Y., Xiong G., Yao S. (2019). Psychometric Properties of the Chinese Version of the Childhood Trauma Questionnaire-Short Form (CTQ-SF) among Undergraduates and Depressive Patients. Child Abuse Negl..

[77] Kim D., Bae H., Han C., Oh H.Y., MacDonald K. (2013). Psychometric Properties of the Childhood Trauma Questionnaire-Short Form (CTQ-SF) in Korean Patients with Schizophrenia. Schizophr. Res..

[78] Mokkink L.B., Terwee C.B., Patrick D.L., Alonso J., Stratford P.W., Knol D.L., Bouter L.M., de Vet H.C.W. (2010). The COSMIN Checklist for Assessing the Methodological Quality of Studies on Measurement Properties of Health Status Measurement Instruments: An International Delphi Study. Qual. Life Res..

[79] Zhu J., Lowen S.B., Anderson C.M., Ohashi K., Khan A., Teicher M.H. (2019). Association of Prepubertal and Postpubertal Exposure to Childhood Maltreatment with Adult Amygdala Function. JAMA Psychiatry.

[80] Schalinski I., Teicher M.H., Nischk D., Hinderer E., Müller O., Rockstroh B. (2016). Type and Timing of Adverse Childhood Experiences Differentially Affect Severity of PTSD, Dissociative and Depressive Symptoms in Adult Inpatients. BMC Psychiatry.

[81] Schoedl A.F., Costa M.C.P., Mari J.J., Mello M.F., Tyrka A.R., Carpenter L.L., Price L.H. (2010). The Clinical Correlates of Reported Childhood Sexual Abuse: An Association between Age at Trauma Onset and Severity of Depression and PTSD in Adults. J. Child Sex. Abuse.

[82] Isele D., Teicher M.H., Ruf-Leuschner M., Elbert T., Kolassa I.-T., Schury K., Schauer M. (2014). KERF–Ein Instrument Zur Umfassenden Ermittlung Belastender Kindheitserfahrungen. Z. Klin. Psychol. Psychother..

[83] Hawker D.S.J., Boulton M.J. (2000). Twenty Years’ Research on Peer Victimization and Psychosocial Maladjustment: A Meta-Analytic Review of Cross-Sectional Studies. J. Child Psychol. Psychiatry.

[84] Teicher M.H., Vitaliano G.D. (2011). Witnessing Violence toward Siblings: An Understudied but Potent Form of Early Adversity. PLoS ONE.

