# Supplementary material for: The Suitability of the Childhood Trauma Questionnaire in Criminal Offender Samples
Source: Int J Environ Res Public Health. 2023 Mar 15;20(6):5195. doi: 10.3390/ijerph20065195 (PMC10048956; doi:10.3390/ijerph20065195)
Supplement: Supplementary file 1 [file ijerph-20-05195-s001.zip › ijerph-2253846-supplementary/Table S1.docx]

## Table S1. Numbers of Lifetime Mental Disorders Depending on the Context of Evaluation.

| **Type of mental disorder** | **Criminal resposibility**  **(*n* = 131)** | **Risk assessment**  **(*n* = 100)** | **χ²** | ***p*** | **φ** |
| --- | --- | --- | --- | --- | --- |
| Schizophrenia or other psychotic disorders | 14 | 2 | 5.36 | 0.021 | -0.17 |
| Affective disorders  (depression, bipolar) | 29 | 4 | 13.79 | <0.001 | -0.26 |
|  |  |  |  |  |  |
| Anxiety, obsessive compulsive disorder, or  adjustment disorder | 17 | 3 | 5.93 | 0.015 | -0.18 |
| Personality disorders | 33 | 16 | 2.34 | 0.126 | -0.11 |
| Paraphilic disorders | 3 | 6 | 1.21 | 0.271 | 0.09 |
| ADHD or conduct disorder | 29 | 8 | 7.41 | 0.006 | -0.19 |
| Substance use disorders | 84 | 27 | 29.84 | <0.001 | -0.37 |
| Post-traumatic stress disorder | 3 | 1 | 0.06 | 0.814 | -0.05 |
