# Supplementary material for: The Suitability of the Childhood Trauma Questionnaire in Criminal Offender Samples
Source: Int J Environ Res Public Health. 2023 Mar 15;20(6):5195. doi: 10.3390/ijerph20065195 (PMC10048956; doi:10.3390/ijerph20065195)
Supplement: Supplementary file 1 [file ijerph-20-05195-s001.zip › ijerph-2253846-supplementary/Table S2.docx]

## Table S2. Cronbach´s α of the Childhood Trauma Questionnaire Depending on the Context of Evaluation.

| **CTQ-SF** | **Cronbach‘s α** | | | |
| --- | --- | --- | --- | --- |
|  | **Criminal responsibility (*n* = 131)** | | **Risk assessment (*n* = 100)** | |
|  | **self** | **external** | **self** | **external** |
| Sum | 0.94 | 0.94 | 0.97 | 0.97 |
| EA | 0.93 | 0.94 | 0.95 | 0.97 |
| PA | 0.92 | 0.90 | 0.95 | 0.91 |
| SA | 0.94 | 0.92 | 0.97 | 0.93 |
| EN | 0.96 | 0.98 | 0.97 | 0.99 |
| PN | 0.73 | 0.73 | 0.76 | 0.80 |
| Note. self = self assessment, external = external assessment, Sum = Sum score, EA = emotional abuse, PA = physical abuse, SA = sexual abuse, EN = emotional neglect, PN = physical neglect. Tests were conducted one-sided. | | | | |
