# Supplementary material for: The Suitability of the Childhood Trauma Questionnaire in Criminal Offender Samples
Source: Int J Environ Res Public Health. 2023 Mar 15;20(6):5195. doi: 10.3390/ijerph20065195 (PMC10048956; doi:10.3390/ijerph20065195)
Supplement: Supplementary file 1 [file ijerph-20-05195-s001.zip › ijerph-2253846-supplementary/Table S3.docx]

## Table S3. Correlations of Childhood Trauma Questionnaire Self and External Assessment in the Total Sample (*N* = 231).

|  | CTQ-SF | M | SD | 1 | 2 | 3 | 4 | 5 | 6 | 7 | 8 | 9 | 10 | 11 |
| --- | --- | --- | --- | --- | --- | --- | --- | --- | --- | --- | --- | --- | --- | --- |
|  | Self assessment |  |  |  |  |  |  |  |  |  |  |  |  |  |
| 1 | Sum | 44.41 | 19.00 |  |  |  |  |  |  |  |  |  |  |  |
| 2 | EA | 9.30 | 5.18 | 0.92**  [0.89, 0.94] |  |  |  |  |  |  |  |  |  |  |
| 3 | PA | 8.16 | 4.94 | 0.87**  [0.84, 0.90] | 0.80**  [0.75, 0.84] |  |  |  |  |  |  |  |  |  |
| 4 | SA | 5.81 | 2.86 | 0.48**  [0.38, 0.58] | 0.35**  [0.23, 0.46] | 0.42**  [0.30, 0.52] |  |  |  |  |  |  |  |  |
| 5 | EN | 11.50 | 6.02 | 0.90**  [0.87, 0.92] | 0.79**  [0.73, 0.83] | 0.68**  [0.60, 0.74] | 0.27**  [0.14, 0.38] |  |  |  |  |  |  |  |
| 6 | PN | 9.65 | 4.02 | 0.79**  [0.73, 0.83] | 0.63**  [0.55, 0.71] | 0.56**  [0.46, 0.64] | 0.21**  [0.08, 0.33] | 0.71**  [0.64, 0.77] |  |  |  |  |  |  |
|  |  |  |  |  |  |  |  |  |  |  |  |  |  |  |
|  | External assessment |  |  |  |  |  |  |  |  |  |  |  |  |  |
| 7 | Sum | 40.62 | 17.38 | 0.80**  [0.75, 0.84] | 0.74**  [0.68, 0.80] | 0.69**  [0.62, 0.75] | 0.42**  [0.31, 0.53] | 0.69**  [0.61, 0.75] | 0.63**  [0.54, 0.70] |  |  |  |  |  |
| 8 | EA | 8.16 | 4.91 | 0.70**  [0.63, 0.76] | 0.68**  [0.61, 0.75] | 0.65**  [0.56, 0.71] | 0.30**  [0.18, 0.41] | 0.59**  [0.50, 0.67] | 0.55**  [0.45, 0.63] | 0.91**  [0.89, 0.93] |  |  |  |  |
| 9 | PA | 7.18 | 4.07 | 0.68**  [0.60, 0.74] | 0.65**  [0.57, 0.72] | 0.70**  [0.62, 0.76] | 0.30**  [0.18, 0.42] | 0.54**  [0.44, 0.62] | 0.49**  [0.38, 0.58] | 0.83**  [0.79, 0.87] | 0.80**  [0.75, 0.84] |  |  |  |
| 10 | SA | 5.55 | 2.25 | 0.41**  [0.30, 0.51] | 0.30**  [0.18, 0.41] | 0.27**  [0.14, 0.38] | 0.81**  [0.76, 0.85] | 0.27**  [0.15, 0.39] | 0.25**  [0.13, 0.37] | 0.51**  [0.40, 0.60] | 0.36**  [0.24, 0.46] | 0.37**  [0.25, 0.47] |  |  |
| 11 | EN | 11.71 | 5.84 | 0.74**  [0.68, 0.79] | 0.69**  [0.62, 0.75] | 0.62**  [0.53, 0.69] | 0.29**  [0.17, 0.41] | 0.70**  [0.63, 0.76] | 0.60**  [0.51, 0.67] | 0.90**  [0.88, 0.93] | 0.76**  [0.70, 0.81] | 0.62**  [0.53, 0.69] | 0.33**  [0.21, 0.44] |  |
| 12 | PN | 8.01 | 3.62 | 0.67**  [0.59, 0.73] | 0.61**  [0.52, 0.69] | 0.51**  [0.41, 0.60] | 0.31**  [0.19, 0.43] | 0.60**  [0.52, 0.68] | 0.61**  [0.53, 0.69] | 0.85**  [0.82, 0.89] | 0.69**  [0.61, 0.75] | 0.57**  [0.48, 0.65] | 0.38**  [0.26, 0.48] | 0.80**  [0.75, 0.84] |
|  |  |  |  |  |  |  |  |  |  |  |  |  |  |  |
| Note. M *=* Mean, SD = Standard deviation, Sum = Sum score, EA = emotional abuse, PA = physical abuse, SA = sexual abuse, EN = emotional neglect, PN = physical neglect. Values in square brackets indicate the 95% confidence interval for each correlation. Tests were conducted two-sided.  * *p* < 0.05, ** *p* < 0.01. | | | | | | | | | | | | | | |
