# Supplementary material for: The Suitability of the Childhood Trauma Questionnaire in Criminal Offender Samples
Source: Int J Environ Res Public Health. 2023 Mar 15;20(6):5195. doi: 10.3390/ijerph20065195 (PMC10048956; doi:10.3390/ijerph20065195)
Supplement: Supplementary file 1 [file ijerph-20-05195-s001.zip › ijerph-2253846-supplementary/Table S4.docx]

## Table S4. Correlations of Childhood Trauma Questionnaire Self and External Assessment in Offenders Evaluated for Criminal Responsibility (*n* = 131).

|  | CTQ-SF | M | SD | 1 | 2 | 3 | 4 | 5 | 6 | 7 | 8 | 9 | 10 | 11 |
| --- | --- | --- | --- | --- | --- | --- | --- | --- | --- | --- | --- | --- | --- | --- |
|  | Self assessment |  |  |  |  |  |  |  |  |  |  |  |  |  |
| 1 | Sum | 43.26 | 16.64 |  |  |  |  |  |  |  |  |  |  |  |
| 2 | EA | 9.02 | 4.81 | 0.89**  [0.85, 0.92] |  |  |  |  |  |  |  |  |  |  |
| 3 | PA | 7.55 | 4.26 | 0.83**  [0.76, 0.87] | 0.76**  [0.67, 0.82] |  |  |  |  |  |  |  |  |  |
| 4 | SE | 5.56 | 2.00 | 0.28**  [0.11, 0.43] | 0.13  [-0.04, 0.29] | 0.15  [-0.02, 0.32] |  |  |  |  |  |  |  |  |
| 5 | EN | 11.54 | 5.74 | 0.90**  [0.86, 0.93] | 0.75**  [0.67, 0.82] | 0.62**  [0.50, 0.72] | 0.17  [-0.00, 0.33] |  |  |  |  |  |  |  |
| 6 | PN | 9.60 | 4.02 | 0.77**  [0.69, 0.83] | 0.56**  [0.43, 0.67] | 0.49**  [0.35, 0.61] | 0.10  [-0.08, 0.26] | 0.66**  [0.56, 0.75] |  |  |  |  |  |  |
|  |  |  |  |  |  |  |  |  |  |  |  |  |  |  |
|  | External assessment |  |  |  |  |  |  |  |  |  |  |  |  |  |
| 7 | Sum | 39.34 | 14.11 | 0.74**  [0.65, 0.81] | 0.68**  [0.58, 0.76] | 0.61**  [0.49, 0.71] | 0.25**  [0.08, 0.40] | 0.62**  [0.51, 0.72] | 0.58**  [0.46, 0.69] |  |  |  |  |  |
| 8 | EA | 7.60 | 3.97 | 0.63**  [0.52, 0.73] | 0.64**  [0.53, 0.73] | 0.57**  [0.44, 0.67] | 0.14  [-0.03, 0.30] | 0.50**  [0.36, 0.62] | 0.47**  [0.32, 0.59] | 0.89**  [0.85, 0.92] |  |  |  |  |
| 9 | PA | 6.63 | 3.23 | 0.62**  [0.50, 0.71] | 0.60**  [0.48, 0.70] | 0.64**  [0.52, 0.73] | 0.06  [-0.11, 0.23] | 0.48**  [0.33, 0.60] | 0.44**  [0.29, 0.57] | 0.75**  [0.67, 0.82] | 0.74**  [0.65, 0.81] |  |  |  |
| 10 | SA | 5.34 | 1.59 | 0.21*  [0.04, 0.37] | 0.05  [-0.12, 0.22] | 0.05  [-0.13, 0.22] | 0.88**  [0.83, 0.91] | 0.15  [-0.02, 0.32] | 0.10  [-0.07, 0.27] | 0.24**  [0.07, 0.40] | 0.10  [-0.07, 0.27] | 0.02  [-0.15, 0.19] |  |  |
| 11 | EN | 11.79 | 5.47 | 0.67**  [0.56, 0.75] | 0.59**  [0.47, 0.70] | 0.52**  [0.38, 0.64] | 0.16  [-0.01, 0.32] | 0.62**  [0.50, 0.72] | 0.53**  [0.40, 0.64] | 0.89**  [0.85, 0.92] | 0.67**  [0.57, 0.76] | 0.49**  [0.35, 0.61] | 0.16  [-0.01, 0.32] |  |
| 12 | PN | 7.98 | 3.33 | 0.58**  [0.46, 0.69] | 0.53**  [0.40, 0.65] | 0.42**  [0.27, 0.55] | 0.14  [-0.03, 0.30] | 0.49**  [0.35, 0.61] | 0.57**  [0.44, 0.67] | 0.87**  [0.82, 0.90] | 0.70**  [0.60, 0.78] | 0.51**  [0.38, 0.63] | 0.15  [-0.03, 0.31] | 0.77**  [0.69, 0.83] |
|  |  |  |  |  |  |  |  |  |  |  |  |  |  |  |
| *Note. M =* Mean, SD = Standard deviation, Sum = Sum score, EA = emotional abuse, PA = physical abuse, SA = sexual abuse, EN = emotional neglect, PN = physical neglect. Values in square brackets indicate the 95% confidence interval for each correlation. Tests were conducted two-sided.  * *p* < 0.05, ** *p* < 0.01. | | | | | | | | | | | | | | |
