# Supplementary material for: The Suitability of the Childhood Trauma Questionnaire in Criminal Offender Samples
Source: Int J Environ Res Public Health. 2023 Mar 15;20(6):5195. doi: 10.3390/ijerph20065195 (PMC10048956; doi:10.3390/ijerph20065195)
Supplement: Supplementary file 1 [file ijerph-20-05195-s001.zip › ijerph-2253846-supplementary/Table S5.docx]

## Table S5. Correlations of Childhood Trauma Questionnaire Self and External Assessment in Offenders Evaluated for Risk Assessment (*n* = 100).

|  | CTQ-SF | M | SD | 1 | 2 | 3 | 4 | 5 | 6 | 7 | 8 | 9 | 10 | 11 |
| --- | --- | --- | --- | --- | --- | --- | --- | --- | --- | --- | --- | --- | --- | --- |
|  | Self assessment |  |  |  |  |  |  |  |  |  |  |  |  |  |
| 1 | Sum | 45.92 | 21.71 |  |  |  |  |  |  |  |  |  |  |  |
| 2 | EA | 9.68 | 5.64 | 0.94**  [0.91, 0.96] |  |  |  |  |  |  |  |  |  |  |
| 3 | PA | 8.95 | 5.64 | 0.91**  [0.87, 0.94] | 0.84**  [0.77, 0.89] |  |  |  |  |  |  |  |  |  |
| 4 | SA | 6.14 | 3.68 | 0.60**  [0.46, 0.71] | 0.49**  [0.33, 0.63] | 0.55**  [0.40, 0.68] |  |  |  |  |  |  |  |  |
| 5 | EN | 11.44 | 6.39 | 0.90**  [0.86, 0.93] | 0.83**  [0.75, 0.88] | 0.74**  [0.64, 0.82] | 0.35**  [0.16, 0.51] |  |  |  |  |  |  |  |
| 6 | PN | 9.71 | 4.05 | 0.82**  [0.74, 0.87] | 0.72**  [0.61, 0.80] | 0.64**  [0.51, 0.75] | 0.30**  [0.11, 0.47] | 0.76**  [0.66, 0.83] |  |  |  |  |  |  |
|  |  |  |  |  |  |  |  |  |  |  |  |  |  |  |
|  | External assessment |  |  |  |  |  |  |  |  |  |  |  |  |  |
| 7 | Sum | 42.29 | 20.88 | 0.84**  [0.77, 0.89] | 0.80**  [0.71, 0.86] | 0.74**  [0.64, 0.82] | 0.51**  [0.35, 0.64] | 0.76**  [0.66, 0.83] | 0.69**  [0.58, 0.78] |  |  |  |  |  |
| 8 | EA | 8.90 | 5.85 | 0.75**  [0.65, 0.82] | 0.72**  [0.61, 0.80] | 0.69**  [0.57, 0.78] | 0.37**  [0.19, 0.53] | 0.68**  [0.56, 0.78] | 0.64**  [0.51, 0.75] | 0.93**  [0.89, 0.95] |  |  |  |  |
| 9 | PA | 7.90 | 4.88 | 0.72**  [0.61, 0.80] | 0.69**  [0.58, 0.78] | 0.73**  [0.62, 0.81] | 0.40**  [0.22, 0.55] | 0.61**  [0.47, 0.72] | 0.56**  [0.40, 0.68] | 0.88**  [0.83, 0.92] | 0.83**  [0.75, 0.88] |  |  |  |
| 10 | SA | 5.83 | 2.89 | 0.53**  [0.37, 0.65] | 0.45**  [0.28, 0.60] | 0.38**  [0.19, 0.53] | 0.78**  [0.69, 0.85] | 0.37**  [0.18, 0.53] | 0.38**  [0.20, 0.54] | 0.63**  [0.50, 0.74] | 0.47**  [0.30, 0.61] | 0.52**  [0.36, 0.65] |  |  |
| 11 | EN | 11.60 | 6.31 | 0.81**  [0.73, 0.87] | 0.79**  [0.70, 0.85] | 0.72**  [0.60, 0.80] | 0.40**  [0.22, 0.55] | 0.78**  [0.69, 0.85] | 0.67**  [0.55, 0.77] | 0.94**  [0.91, 0.96] | 0.85**  [0.78, 0.90] | 0.74**  [0.64, 0.82] | 0.46**  [0.29, 0.60] |  |
| 12 | PN | 8.06 | 3.99 | 0.74**  [0.64, 0.82] | 0.69**  [0.57, 0.78] | 0.59**  [0.45, 0.71] | 0.43**  [0.25, 0.58] | 0.72**  [0.61, 0.80] | 0.67**  [0.54, 0.76] | 0.86**  [0.79, 0.90] | 0.69**  [0.57, 0.78] | 0.63**  [0.50, 0.74] | 0.53**  [0.37, 0.66] | 0.83**  [0.75, 0.88] |
|  |  |  |  |  |  |  |  |  |  |  |  |  |  |  |
| Note. M *=* Mean, SD = Standard deviation, Sum = Sum score, EA = emotional abuse, PA = physical abuse, SA = sexual abuse, EN = emotional neglect, PN = physical neglect. Values in square brackets indicate the 95% confidence interval for each correlation. Tests were conducted two-sided.  * *p* < 0.05, ** *p* < 0.01. | | | | | | | | | | | | | | |
