# Supplementary material for: The Suitability of the Childhood Trauma Questionnaire in Criminal Offender Samples
Source: Int J Environ Res Public Health. 2023 Mar 15;20(6):5195. doi: 10.3390/ijerph20065195 (PMC10048956; doi:10.3390/ijerph20065195)
Supplement: Supplementary file 1 [file ijerph-20-05195-s001.zip › ijerph-2253846-supplementary/Table S6.docx]

## Tables S6. Intra-class Correlation Coefficient between Self and External Assessment within the Groups Criminal Responsibility and Risk Assessment.

**Table S6.1.** Absolute Agreement between CTQ Self- and External Assessment within the Criminal Responsibility Group.

|  |  | 95% CI | | F-Test with True Value 0 | | | |
| --- | --- | --- | --- | --- | --- | --- | --- |
| CTQ-SF | **ICC** | **Lower limit** | **Upper limit** | ***F*** | ***df1*** | ***df2*** | ***p*** |
| Sum | 0.71 | 0.59 | 0.79 | 6.40 | 130 | 130 | <0.001 |
| EA | 0.60 | 0.45 | 0.71 | 4.40 | 130 | 130 | <0.001 |
| PA | 0.60 | 0.47 | 0.70 | 4.20 | 130 | 130 | <0.001 |
| SA | 0.85 | 0.79 | 0.89 | 12.70 | 130 | 130 | <0.001 |
| EN | 0.62 | 0.50 | 0.72 | 4.30 | 130 | 130 | <0.001 |
| PN | 0.51 | 0.31 | 0.65 | 3.50 | 130 | 130 | <0.001 |
| Note. *n* = 131, Number of judges = 2. Sum = sum score, EA = emotional abuse, PA = physical abuse, SA = sexual abuse, EN = emotional neglect, PN = physical neglect. | | | | | | | |

**Table S6.2.** Absolute Agreement between CTQ Self- and External Assessment within the Risk Assessment Group.

|  |  | 95% CI | | F-Test with True Value 0 | | | |
| --- | --- | --- | --- | --- | --- | --- | --- |
| CTQ-SF | **ICC** | **Lower limit** | **Upper limit** | ***F*** | ***df1*** | ***df2*** | ***p*** |
| Sum | 0.83 | 0.74 | 0.88 | 11.40 | 100 | 100 | <0.001 |
| EA | 0.71 | 0.60 | 0.80 | 6.10 | 100 | 100 | <0.001 |
| PA | 0.71 | 0.59 | 0.79 | 6.10 | 100 | 100 | <0.001 |
| SA | 0.76 | 0.66 | 0.83 | 7.20 | 100 | 100 | <0.001 |
| EN | 0.78 | 0.69 | 0.85 | 8.10 | 100 | 100 | <0.001 |
| PN | 0.62 | 0.40 | 0.75 | 5.00 | 100 | 100 | <0.001 |
| Note. *n* = 100, Number of judges = 2. Sum = sum score, EA = emotional abuse, PA = physical abuse, SA = sexual abuse, EN = emotional neglect, PN = physical neglect. | | | | | | | |
