# Supplementary material for: The Suitability of the Childhood Trauma Questionnaire in Criminal Offender Samples
Source: Int J Environ Res Public Health. 2023 Mar 15;20(6):5195. doi: 10.3390/ijerph20065195 (PMC10048956; doi:10.3390/ijerph20065195)
Supplement: Supplementary file 1 [file ijerph-20-05195-s001.zip › ijerph-2253846-supplementary/Table S7.docx]

## Tables S7. Stepwise Linear Regression with Interaction to Predict the External Assessment of the Childhood Trauma Questionnaire.

**Table S7.1.** Stepwise Linear Regression with Interaction to Predict the CTQ-External Assessment of the Sum Score.

|  | **R^2^** | ***p*** | **Predictor** | **Estimate** | **SE** | **95% CI** | | ***p*** |
| --- | --- | --- | --- | --- | --- | --- | --- | --- |
|  |  |  |  |  |  | **LL** | **UL** |  |
|  | 0.637 | <0.001 | CTQ-self Sum | 0.730 | 0.036 | 0.65 | 0.80 | <0.001 |
| Model a | 0.638 | 0.472 | CTQ-self Sum | 0.728 | 0.037 | 0.66 | 0.80 | <0.001 |
|  |  |  | CE | 1.009 | 1.399 | -1.75 | 3.77 | 0.472 |
| Model b | 0.647 | 0.015 | CTQ-self Sum | 0.627 | 0.055 | 0.52 | 0.74 | <0.001 |
|  |  |  | CE | 1.008 | 1.384 | -1.72 | 3.73 | 0.467 |
|  |  |  | CTQ-self Sum × CE | 0.180 | 0.073 | 0.04 | 0.32 | 0.015 |
| Model c^1^ | 0.649 | 0.830 | CTQ-self Sum | 0.629 | 0.055 | 0.52 | 0.74 | <0.001 |
|  |  |  | CE | 0.643 | 1.450 | -2.21 | 3.50 | 0.658 |
|  |  |  | CTQ-self Sum × CE | 0.176 | 0.074 | 0.03 | 0.32 | 0.018 |
| Note. SE = standard error, CI = confidence interval, LL = lower limit, UL = upper limit, self = self-assessment, Sum = sum score, CE = context of evaluation.  ^1^Analyses controlled for age and intelligence. | | | | | | | | |

**Table S7.2.** Stepwise Linear Regression with Interaction to Predict CTQ-External Assessment of the EA-subscale.

|  | **R^2^** | ***p*** | **Predictor** | **Estimate** | **SE** | **95% CI** | | ***p*** |
| --- | --- | --- | --- | --- | --- | --- | --- | --- |
|  |  |  |  |  |  | **LL** | **UL** |  |
|  | 0.466 | <0.001 | CTQ-self EA | 0.647 | 0.046 | 0.56 | 0.74 | <0.001 |
| Model a | 0.474 | 0.069 | CTQ-self EA | 0.642 | 0.046 | 0.55 | 0.73 | <0.001 |
|  |  |  | CE | 0.870 | 0.476 | -0.07 | 1.81 | 0.069 |
| Model b | 0.487 | 0.018 | CTQ-self EA | 0.532 | 0.065 | 0.41 | 0.66 | <0.001 |
|  |  |  | CE | 0.862 | 0.471 | -0.07 | 1.79 | 0.068 |
|  |  |  | CTQ-self EA × CE | 0.215 | 0.090 | 0.04 | 0.39 | 0.018 |
| Model c^1^ | 0.492 | 0.547 | CTQ-self EA | 0.537 | 0.065 | 0.41 | 0.67 | <0.001 |
|  |  |  | CE | 0.662 | 0.492 | -0.31 | 1.63 | 0.180 |
|  |  |  | CTQ-self EA × CE | 0.209 | 0.091 | 0.03 | 0.39 | .023 |
| Note. SE = standard error, CI = confidence interval, LL = lower limit, UL = upper limit, self = self-assessment, EA = emotional abuse, CE = context of evaluation.  ^1^Analyses controlled for age and intelligence. | | | | | | | | |

**Table S7.3.** Stepwise Linear Regression with Interaction to Predict the CTQ-External Assessment of the PA-subscale.

|  | **R^2^** | ***p*** | **Predictor** | **Estimate** | **SE** | **95% CI** | | ***p*** |
| --- | --- | --- | --- | --- | --- | --- | --- | --- |
|  |  |  |  |  |  | **LL** | **UL** |  |
|  | 0.485 | <0.001 | CTQ-self PA | 0.573 | 0.039 | 0.50 | 0.65 | <0.001 |
| Model a | 0.488 | 0.228 | CTQ-self PA | 0.566 | 0.039 | 0.49 | 0.64 | <0.001 |
|  |  |  | CE | 0.474 | 0.392 | -0.30 | 1.25 | 0.228 |
| Model b | 0.495 | 0.070 | CTQ-self PA | 0.484 | 0.060 | 0.37 | 0.60 | <0.001 |
|  |  |  | CE | 0.475 | 0.390 | -0.29 | 1.24 | 0.225 |
|  |  |  | CTQ-self PA × CE | 0.144 | 0.079 | -0.01 | 0.30 | 0.070 |
| Model c^1^ | 0.504 | 0.290 | CTQ-self PA | 0.485 | 0.060 | 0.37 | 0.60 | <0.001 |
|  |  |  | CE | 0.261 | 0.406 | -0.54 | 1.06 | 0.520 |
|  |  |  | CTQ-self PA × CE | 0.139 | 0.080 | -0.02 | 0.30 | 0.083 |
| Note. SE = standard error, CI = confidence interval, LL = lower limit, UL = upper limit, self = self-assessment, PA = physical abuse, CE = context of evaluation.  ^1^Analyses controlled for age and intelligence. | | | | | | | | |

**Table S7.4.** Stepwise Linear Regression with Interaction to Predict the CTQ-External Assessment of the SA-subscale.

|  | **R^2^** | ***p*** | **Predictor** | **Estimate** | **SE** | **95% CI** | | ***p*** |
| --- | --- | --- | --- | --- | --- | --- | --- | --- |
|  |  |  |  |  |  | **LL** | **UL** |  |
|  | 0.654 | <.0001 | CTQ-self SA | 0.638 | 0.031 | 0.58 | 0.70 | <0.001 |
| Model a | 0.654 | 0.514 | CTQ-self SA | 0.636 | 0.031 | 0.58 | 0.70 | <0.001 |
|  |  |  | CE | 0.116 | 0.178 | -0.23 | 0.47 | 0.514 |
| Model b | 0.656 | 0.226 | CTQ-self SA | 0.700 | 0.058 | 0.58 | 0.81 | <0.001 |
|  |  |  | CE | 0.120 | 0.178 | -0.24 | 0.46 | 0.514 |
|  |  |  | CTQ-self SA × CE | -0.083 | 0.069 | -0.22 | 0.05 | 0.226 |
| Model c^1^ | 0.659 | 0.643 | CTQ-self SA | 0.696 | 0.059 | 0.58 | 0.81 | <0.001 |
|  |  |  | CE | 0.099 | 0.185 | -0.27 | 0.46 | 0.596 |
|  |  |  | CTQ-self SA × CE | -0.085 | 0.069 | -0.22 | 0.05 | 0.218 |
| Note. SE = standard error, CI = confidence interval, LL = lower limit, UL = upper limit, SE = self-assessment, SA = sexual abuse, CE = context of evaluation. ^1^Analyses controlled for age and intelligence. | | | | | | | | |

**Table S7.5.** Stepwise Linear Regression with Interaction to Predict the CTQ-External Assessment of the EN-subscale.

|  | **R^2^** | ***p*** | **Prädiktor** | **Estimate** | **SE** | **95% CI** | | ***p*** |
| --- | --- | --- | --- | --- | --- | --- | --- | --- |
|  |  |  |  |  |  | **LL** | **UL** |  |
|  | 0.489 | <0.001 | CTQ-self EN | 0.679 | 0.046 | 0.59 | 0.77 | <0.001 |
| Model a | 0.489 | 0.834 | CTQ-self EN | 0.679 | 0.046 | 0.59 | 0.77 | <0.001 |
|  |  |  | CE | -0.117 | 0.557 | -1.21 | 0.98 | 0.834 |
| Model b | 0.497 | 0.053 | CTQ-self EN | 0.592 | 0.064 | 0.47 | 0.72 | <0.001 |
|  |  |  | CE | -0.116 | 0.553 | -1.21 | 0.98 | 0.835 |
|  |  |  | CTQ-self EN × CE | 0.178 | 0.091 | 0.00 | 0.36 | 0.053 |
| Model c^1^ | 0.499 | 0.876 | CTQ-self EN | 0.594 | 0.064 | 0.47 | 0.72 | <0.001 |
|  |  |  | CE | -0.162 | 0.580 | -1.31 | 0.98 | 0.780 |
|  |  |  | CTQ-self EN × CE | 0.177 | 0.092 | -0.01 | 0.36 | 0.057 |
| Note. SE = standard error, CI = confidence interval, LL = lower limit, UL = upper limit, SE = self-assessment, EN = emotional neglect, CE = context of evaluation. ^1^Analyses controlled for age and intelligence. | | | | | | | | |

**Table S7.6.** Stepwise Linear Regression with Interaction to Predict the CTQ-External Assessment of the PN-subscale.

|  | **R^2^** | ***p*** | **Predictor** | **Estimate** | **SE** | **95% CI** | | ***p*** |
| --- | --- | --- | --- | --- | --- | --- | --- | --- |
|  |  |  |  |  |  | **LL** | **UL** |  |
|  | 0.376 | <0.001 | CTQ-self PN | 0.552 | 0.047 | 0.46 | 0.65 | <0.001 |
| Model a | 0.376 | 0.959 | CTQ-self PN | 0.552 | 0.047 | 0.46 | 0.65 | <0.001 |
|  |  |  | CE | 0.020 | 0.382 | -0.73 | 0.77 | 0.959 |
| Model b | 0.387 | 0.052 | CTQ-self PN | 0.471 | 0.062 | 0.35 | 0.59 | <0.001 |
|  |  |  | CE | 0.017 | 0.379 | -0.73 | 0.76 | 0.965 |
|  |  |  | CTQ-self PN × CE | 0.185 | 0.094 | 0.00 | 0.37 | 0.052 |
| Model c^1^ | 0.389 | 0.845 | CTQ-self PN | 0.473 | 0.063 | 0.35 | 0.60 | <0.001 |
|  |  |  | CE | 0.105 | 0.397 | -0.68 | 0.89 | 0.792 |
|  |  |  | CTQ-self PN × CE | 0.185 | 0.097 | -0.01 | 0.38 | 0.056 |
| Note. SE = standard error, CI = confidence interval, LL = lower limit, UL = upper limit, self = self-assessment, PN = physical neglect, CE = context of evaluation. ^1^Analyses controlled for age and intelligence. | | | | | | | | |
